# Educational environment perceptions across curriculum formats: structural associations among DREEM domains and academic self-perceptions

**DOI:** 10.3389/fmed.2026.1879003

**Published:** 2026-09-24

**Authors:** Burhanettin Ozdemir, Abdul Ahad Shaikh, Muhammad Atif Mazhar, Sadia Qazi

**Affiliations:** 1 College of Business, Alfaisal University, Riyadh, Saudi Arabia; 2 Department of Physiology, College of Medicine, Alfaisal University, Riyadh, Saudi Arabia; 3 Department of Anatomy, College of Medicine, Alfaisal University, Riyadh, Saudi Arabia

**Keywords:** DREEM, educational environment, medical education, psychometric validation, structural equation modeling, team-based learning

## Abstract

**Introduction:**

Team-Based Learning (TBL) is increasingly used in health professions education; however, the Dundee Ready Education Environment Measure (DREEM) has rarely been formally evaluated in TBL-based medical curricula. This study examined the structural validity of the DREEM, assessed whether associations with academic self-perceptions differed by year and gender, tested an indirect pathway between learning quality and atmosphere, and evaluated group mean differences.

**Methods:**

A cross-sectional survey was administered to 411 Year 1 and Year 2 medical students at the Alfaisal University College of Medicine, Riyadh, Saudi Arabia. Analyses included confirmatory factor analysis using weighted least squares mean and variance adjusted estimation, multigroup structural equation modeling, bootstrapped mediation with 5,000 simulations, and multivariate analysis of variance (MANOVA).

**Results:**

Confirmatory factor analysis showed mixed but overall supportive evidence for the five-factor structure (comparative fit index = .996, Tucker–Lewis index = .996, standardized root mean square residual = .086; root mean square error of approximation = .103). The internal consistency coefficients ranged from .643 to .942. Among Year 1 students, academic self-concept was associated with learning and social perceptions, whereas among Year 2 students, all four environmental domains were associated with academic self-concept. Among male students, teacher and social perceptions were more strongly associated with academic self-concept, whereas among female students, learning quality and atmosphere were more strongly associated. Academic self-perceptions showed an indirect association consistent with the partial mediation of the learning–atmosphere relationship. No significant multivariate differences emerged between academic years or genders.

**Discussion:**

For a given institution, these findings support complementing aggregate DREEM scores with subscale and structural analyses to identify cohort-specific patterns across its TBL and TBL + PBL preclinical learning environments.

## Introduction

The educational environment encompasses the social, organizational, and physical conditions within which medical students learn, and it directly influences academic performance, student well-being, and professional development (1, 2). Although curriculum content and faculty expertise are clearly important, the learning climate in which instruction occurs has a significant and often under-recognized impact on students’ academic development. The effect is substantial enough for institutions to systematically assess it using standardized instruments.

Among active, student-centered pedagogical strategies, Team-Based Learning (TBL) has become one of the most widely adopted in health professions education, enabling collaborative interaction, individual responsibility, and the use of knowledge in complex situations in an organized environment of small groups (3). First introduced by Larry Michaelsen as a business learning technique, TBL has been used as a resource-efficient alternative to both conventional lectures and problem-based learning, meeting the demands of large cohorts while preserving the advantages of small group learning (4). The TBL sequence, which includes preparation at the pre-class stage, assurance tests (both individual and team), immediate feedback, and collaborative application, is aimed at making students responsible in their preparation and developing the necessary skills in critical thinking, communication, and teamwork, which are central in clinical practice (3). An umbrella review of 23 systematic reviews, encompassing more than 312 primary studies and over 63,987 participants, found that TBL is widely linked to better performance, increased student engagement, and student satisfaction compared to lecture-based education in health professions education settings (5). A complementary meta-analysis of 33 studies found that TBL students scored significantly higher on post-tests than their lecture-based peers (standardized mean difference 0.96, 95% CI: 0.70 to 1.22), with comparable advantages for knowledge retention and satisfaction (6). Despite this body of outcome evidence, no published study has examined how students perceive the TBL learning environment as a holistic entity using a validated environmental assessment instrument, such as DREEM.

Medical training is psychologically demanding, and the associated strain is linked to higher levels of depression, anxiety, and burnout, with consequences for student well-being (7). The quality of the learning climate has been shown to be related to student engagement and academic achievement (1). These concerns have driven the development of validated instruments for measuring students’ subjective perceptions of their learning environments. The Dundee Ready Education Environment Measure (DREEM), originally developed by Roff et al. (8) through an international Delphi process, is the most widely used instrument in health professions education. It has been translated into over five languages and applied in more than two dozen national contexts (9).

The DREEM has 50 items across five subscales: Students’ Perceptions of Learning (SPL; 12 items), Students’ Perceptions of Teachers (SPT; 11 items), Students’ Academic Self-Perceptions (SASP; 8 items), Students’ Perceptions of Atmosphere (SPA; 12 items), and Students’ Social Self-Perceptions (SSSP; 7 items), yielding a total score of 200, which allows institutions to benchmark their environments and track changes over time.

Over the past decade, DREEM has been applied extensively in Saudi Arabia, Morocco, India, Iran, Syria, Slovenia, Hungary, and the United Kingdom (10–16). This growing body of evidence suggests several recurring patterns, including the relative vulnerability of the social self-perception domain and a tendency for students earlier in training to report more positive perceptions, while also indicating substantial variation by national context, institutional type, academic discipline, and demographic characteristics of the students. However, the DREEM has been used less often in institutions where TBL is the primary pedagogical modality, raising the possibility that TBL-related features such as structured peer interaction, immediate feedback, and collaborative problem-solving may be associated with different perceptions of the learning environment than more traditional instructional approaches. This study examined the TBL learning environment at the College of Medicine, Alfaisal University, using the DREEM inventory to explore environmental strengths, areas of relative weakness, and their possible associations with academic self-perception.

This evidence also suggests that social self-perception often scores lowest across national contexts, and that first-year students tend to rate their environments more favorably than later-year students, although the magnitude of these patterns appears to vary by national context, institutional type, and academic discipline. Notably, published applications of the DREEM in settings where TBL serves as the primary pedagogical model remain limited. This study aims to address this gap.

The present study addresses this gap through four analytical objectives. First, confirmatory factor analysis (CFA) with diagonally weighted least squares (WLSMV) estimation formally tested the five-factor structure of the DREEM against established goodness-of-fit criteria, a step that remains uncommon in the literature despite being a prerequisite for valid subscale interpretation. Second, an analysis of multigroup structural equation modeling (SEM) examines whether the predictive relationships between DREEM environmental subscales and academic self-perception (SASP) differ by academic year and gender, testing whether group membership moderates structural pathways rather than merely score levels. Third, a bootstrapped mediation analysis was conducted to test whether SASP mediates the relationship between learning perceptions (SPL) and atmosphere perceptions (SPA). Fourth, MANOVA formally tests mean-level group differences across subscales and total scores.

### Literature review

The DREEM was developed by Roff et al. (8) through an international Delphi process as a 50-item instrument comprising five theoretically defined dimensions of the educational environment. The instrument has demonstrated strong internal consistency across health profession settings, particularly at the total score level (14, 17), and its subscales provide domain-specific information relevant to educational evaluation. Nevertheless, the factorial structure of the scale has been less consistently examined. Many applications report reliability and descriptive scores without directly testing structural validity (9), although confirmatory evidence has been reported for selected, translated, and modified versions (13, 14). Further evaluation of the original five-factor structure is therefore important when subscale scores are used for group comparisons or in structural analyses.

TBL is particularly relevant to the assessment of educational environments because its instructional design integrates individual preparation, readiness assurance, immediate feedback, and collaborative application (3, 4). Reviews of TBL in health professions education generally report favorable effects on knowledge acquisition, engagement, satisfaction, and collaborative learning, although findings regarding longer-term retention are less consistent (5, 6, 18). Despite the substantial literature on learning outcomes, comparatively little research has examined how students experience the broader educational environment within curricula in which TBL constitutes a major instructional approach.

DREEM studies conducted in Saudi Arabia have demonstrated that students’ perceptions vary across institutions, disciplines, academic stages, and environmental domains. Studies in Riyadh and Jeddah have generally characterized the educational environment as more positive than negative, while identifying variation in learning, academic self-perception, social self-perception, teaching, and atmosphere domains (10, 11, 19–21). Several Saudi studies have also reported differences associated with academic year or gender, although these patterns have not been consistent across settings (19–21). Longitudinal findings further indicate that DREEM profiles can change over time, reinforcing their potential value for monitoring the educational environment rather than relying on a single cross-sectional assessment (21).

International studies have similarly demonstrated substantial contextual variability. Research from Morocco, Syria, Hungary, the United Kingdom, Slovenia, and India has reported differences in total DREEM scores and domain profiles according to institution, discipline, academic stage, and student characteristics (12, 14–17, 22, 23). Social self-perception and aspects of the learning atmosphere have frequently emerged as comparatively weaker domains, although the magnitude and direction of subgroup differences vary considerably. Evidence comparing the DREEM with other learning environment measures also supports the value of examining domain-level patterns rather than relying exclusively on total scores (22). These findings are particularly pertinent to TBL because structured peer interactions and collaborative applications are central elements of its instructional design (24).

Evidence also suggests that DREEM is responsive to institutional and curricular changes. Bakhshialiabad et al. (13) reported improvements across DREEM domains following faculty development interventions and subsequent reassessment, whereas Alshahrani et al. (21) documented declining perceptions across repeated measurement periods. These findings indicate that perceptions of the educational environment are not necessarily static and support the use of structurally sound measures for longitudinal quality monitoring.

Academic self-perception is of particular interest within the DREEM framework because it reflects students’ perceptions of their competence and academic development. Previous studies have identified the SASP as a relatively favorable domain in some settings (10, 11). Within TBL, repeated readiness assurance and collaborative application provide students with structured opportunities to evaluate their understanding in relation to their peers and instructional expectations (4). Therefore, it is theoretically plausible that students who perceive their learning experiences more favorably may report stronger academic self-perceptions, which may, in turn, be associated with their perceptions of the broader learning atmosphere. This provides a rationale for examining the SASP as an intermediate variable in the association between SPL and SPA.

Evidence concerning gender and academic year is inconsistent. Some studies have reported more favorable DREEM perceptions among female students (13, 20), while others have identified higher scores among male students (12) or found no significant gender differences (17, 19). Earlier-year students have tended to report more favorable educational environments in some studies (13, 19, 21), but these analyses have predominantly examined differences in the mean scores. Consequently, relatively little is known about whether the academic year or gender is associated with differences in the relationships among the DREEM domains. Examining these relationships through multigroup structural models may provide information that cannot be obtained from mean comparisons alone.

### Theoretical synthesis and gaps addressed by the present study

The literature reviewed above establishes four consistent empirical patterns that frame the contributions of the present study. First, the SSSP domain universally scored the lowest across national and institutional contexts, indicating that social connectedness is a chronic vulnerability in health professions education, a particularly salient finding given TBL's theoretical emphasis on collaborative peer interaction as a mechanism for improving this domain. Second, early year students consistently rate their environments more favorably, suggesting an erosion of positive perceptions with advancing training that may reflect cumulative workload, clinical disillusionment, or shifting reference standards. Third, the effects of gender on DREEM scores are inconsistent and context-dependent, motivating structural rather than mean-level analysis. Fourth, and most critically, a formal confirmatory psychometric evaluation of the DREEM's five-factor structure is conspicuously absent from most of the existing literature, a gap that undermines the interpretive validity of virtually all comparative and predictive claims built on subscale scores. Compounding these gaps is the near-total absence of DREEM research conducted within TBL-based curricula, leaving educators and institutions without validated, environment-level evidence of how students perceive the holistic educational milieu generated by this increasingly prominent pedagogical approach.

The present study addresses these gaps through a four-pronged analytical strategy: CFA with WLSMV estimation to evaluate structural validity; multigroup SEM to test whether predictive pathways from environmental perceptions to academic self-perceptions differ by year and gender; bootstrapped mediation analysis to examine the mechanism through which learning perceptions influence atmosphere perceptions via academic self-concept; and MANOVA to formally test for group mean differences. By situating its findings within the rich contextual evidence provided by regional and international DREEM research and by focusing specifically on a TBL-centered medical college, the present study contributes to and draws on a growing global literature committed to understanding and improving the medical educational environment.

The present study was guided by the following research questions (RQ):

RQ1. What is the psychometric structure and reliability of the Dundee Ready Education Environment Measure (DREEM) among medical students, as assessed through confirmatory factor analysis?
RQ2. To what extent do the DREEM subscales—Students’ Perceptions of Learning (SPL), Students’ Perceptions of Teachers (SPT), Students’ Academic Self-Perceptions (SASP), Students’ Perceptions of Atmosphere (SPA), and Students’ Social Self-Perceptions (SSSP)—predict students’ academic self-perceptions, and do these relationships differ by academic year and gender?
RQ3. Does academic self-perception (SASP) mediate the relationship between learning perceptions (SPL) and atmosphere perceptions (SPA)?
RQ4. Do the DREEM subscale and total scores differ across academic years (Year 1 vs. Year 2) and gender?

## Methodology

### Participants and procedure

This study employed a cross-sectional survey design. Following ethical approval from the Alfaisal University Institutional Review Board (IRB20290; dated March 19, 2024) and written informed consent from all participants, all Year 1 and Year 2 preclinical medical students enrolled at the Alfaisal University College of Medicine, Riyadh, Saudi Arabia were included. During the 2024-2025 academic year, students were invited to participate.

The two cohorts differed in their predominant pedagogical formats: Year 1 used TBL as the principal instructional approach, whereas Year 2 incorporated both TBL and PBL sessions within a hybrid curriculum. A brief schematic overview of these curricular formats is presented in Figure 1. This distinction provides important context for interpreting the year-stratified analyses, although the academic year and curriculum format cannot be separated as independent effects in the present cross-sectional design.

**Figure 1:**
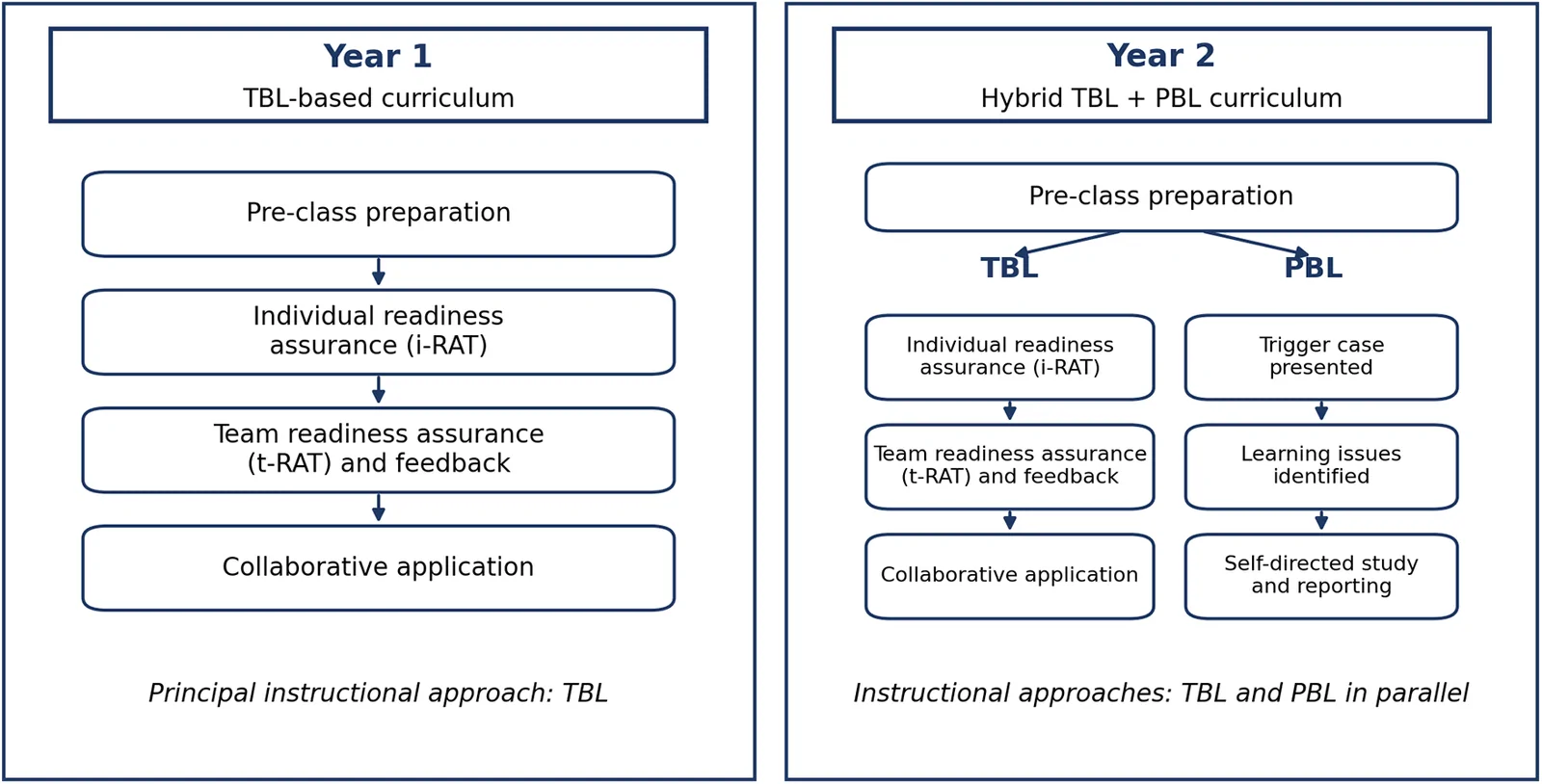
Overview of the pedagogical formats of the two study cohorts.

The survey was administered online through Google Forms sent via students’ emails. At the time of data collection, Year 1 students were completing the Gastrointestinal (GIT) block, and Year 2 students were completing the Neuroscience block. Both groups were surveyed within the same two-week window to minimize intra-year variation related to the academic calendar. Participation was voluntary. No sampling procedure was applied; the entire eligible preclinical population was invited, comprising 700 students (496 in year 1 and 204 in year 2). Because participation was voluntary, the achieved sample size was not fixed in advance, and the precision of the realized sample is reported in the Results.

### Instrument

The present study situates its psychometric evaluation within Kane's (25) argument-based validity framework, treating the CFA results as structural evidence, which is one component of a broader validity argument, alongside internal consistency data as evidence of reliability. Under this framework, the confirmatory factor analysis tests the internal structure inference: whether the five-factor model adequately represents the construct space that the DREEM is intended to measure. The subscale-level prediction of academic self-perceptions (RQ2) and the mediation analysis (RQ3) provide additional evidence pertaining to the relations-to-other-variables inference.

The DREEM inventory has 50 items grouped into five theoretically derived subscales: Students’ Perceptions of Learning (SPL; 12 items), Students’ Perceptions of Teachers (SPT; 11 items), Students’ Academic Self-Perceptions (SASP; 8 items), Students’ Perceptions of Atmosphere (SPA; 12 items), and Students’ Social Self-Perceptions (SSSP; 7 items). The items were rated on a five-point Likert-type scale ranging from 0 (strongly disagree) to 4 (strongly agree), with higher subscale scores indicating more positive perceptions of that environmental dimension. The total scores range from 0 to 200, with published interpretive benchmarks: 0–50, very poor; 51–100, many problems; 101–150, more positive than negative; and 151–200, excellent (26). The DREEM was originally validated by Roff et al. (8) and has demonstrated adequate to excellent reliability across diverse international samples (9, 14). Consistent with the validity argument framing above, the present study treats reliability estimation and structural validity as necessary but insufficient conditions for valid interpretation. Subscale prediction and mediation analyses provide further sources of validity evidence.

### Data analysis

All analyses were performed using R (version 4.5.0) (27). Internal consistency reliability was estimated using Cronbach's alpha for each subscale and the total scale. Consistent with Kane's validity argument framework (25), the analytical strategy was organized to accumulate evidence across multiple inferences: structural evidence (CFA), predictive evidence (multigroup SEM), mechanistic evidence (mediation), and group invariance evidence (MANOVA).

Confirmatory factor analysis (CFA) was conducted using the lavaan package (28) with WLSMV estimation, which is the appropriate estimator for ordinal Likert-type data (29). Model fit was evaluated against conventional thresholds: CFI and TLI ≥ .95, RMSEA ≤ .08, and SRMR ≤ .08 (30).

Prior to testing the structural pathways, measurement invariance was assessed across years (Year 1 vs. Year 2) and gender groups using a sequential approach: configural invariance (same factor structure across groups), metric invariance (equal factor loadings), and scalar invariance (equal item intercepts) (31, 32). The criteria for partial invariance were specified in advance in the event that full invariance was not supported (33). Invariance testing is a prerequisite for meaningful comparisons of latent path coefficients across groups (31, 32).

Bootstrapped mediation analysis was conducted to examine whether the indirect effect of SPL on SPA via SASP was statistically significant, using 5,000 bootstrap simulations with 95% bias-corrected confidence intervals (CIs) (34). Given the cross-sectional design, the findings are interpreted as evidence of an indirect structural pathway rather than an established causal mediation.

Wilcoxon rank-sum tests were used to compare groups based on subscale scores, given the ordinal nature of the Likert-based summed scores. Effect sizes were reported both as Cohen's d, computed from the pooled standard deviation with 95% confidence intervals, and as the rank-biserial correlation (r) derived from the Wilcoxon statistic, which is scale-independent and appropriate for non-parametric comparisons (35). The precision of the achieved sample was estimated *post hoc* as the 95% margin of error for a population proportion (*p* = 0.50), incorporating a finite population correction for the known size of each eligible cohort. Group mean differences across all five DREEM subscales were examined simultaneously using multivariate analysis of variance (MANOVA) with Pillai's trace criterion, which is robust to violations of multivariate normality (36). The significance threshold was set at *α* = .05 for all analyses.

## Results

Of the 700 eligible students invited, 411 completed the survey, yielding an overall response rate of 58.7% (95% margin of error ± 3.1%). Participation was 64.1% in Year 1 (318/496; ±3.3%) and 45.6% in Year 2 (93/204; ±7.5%). The respondents comprised 143 male (34.8%) and 268 female (65.2%) students. Table 1 presents the descriptive statistics for the five DREEM subscales and total score across the full sample, by academic year, and by gender.

**Table 1:** Descriptive statistics for DREEM subscales and total score by full sample, academic year, and gender.

Scale	Full Sample (*N* = 411)		Year 1 (*n* = 318)		Year 2 (*n* = 93)		Male (*n* = 143)		Female (*n* = 268)	
	M	SD	M	SD	M	SD	M	SD	M	SD
SPL	32.04	7.93	32.35	7.43	31.65	8.30	31.25	8.00	32.69	7.39
SPT	29.82	7.35	30.38	6.69	28.55	8.08	29.01	8.00	30.47	6.47
SASP	21.17	5.50	21.17	4.99	21.62	6.32	20.63	5.60	21.61	5.13
SPA	28.06	9.50	28.47	9.06	27.23	10.12	27.57	9.38	28.52	9.28
SSSP	15.80	4.42	15.88	4.10	15.86	4.92	15.75	4.95	15.94	3.90
DREEM Total	126.87	29.11	128.25	26.60	124.90	31.57	124.22	30.40	129.24	26.20

Note. M, mean; SD, standard deviation; SPL, Students’ Perceptions of Learning; SPT, Students’ Perceptions of Teachers; SASP, Students’ Academic Self-Perceptions; SPA, Students’ Perceptions of Atmosphere; SSSP, Students’ Social Self-Perceptions.

The full sample (*N* = 411) had a mean DREEM total score of 126.87 (SD = 29.11, range: 0–192), indicating a moderately positive perception of the educational environment. Among the reported subscale total scores, Students’ Perceptions of Learning (SPL) had the highest raw mean (*M* = 32.04, SD = 7.93), whereas Students’ Social Self-Perceptions (SSSP) had the lowest (*M* = 15.80, SD = 4.42). Year 1 students (*n* = 318) reported slightly higher total scores (*M* = 128.25, SD = 26.60) than Year 2 students (*n* = 93; *M* = 124.90, SD = 31.57). Female students (*n* = 268) also reported slightly higher total scores (*M* = 129.24, SD = 26.20) than male students (*n* = 143; *M* = 124.22, SD = 30.40). Subscale-level patterns were largely consistent across the academic year and gender groups.

### Reliability

Internal consistency reliability estimates were computed for each DREEM subscale and the total score. Cronbach's alpha, as shown in Table 2, varied between.643 (SSSP) and.942 (DREEM total). The total DREEM scale showed excellent reliability (*α* = .942). Subscale alphas for SPL (*α* = .844), SPT (*α* = .842), and SPA (*α* = .871) were in the good range, while SASP (*α* = .795) was acceptable. The SSSP subscale yielded a relatively low alpha of.643, consistent with prior validation studies (37).

**Table 2:** Internal consistency reliability of DREEM subscales.

Subscale	Items (*n*)	Cronbach's *α*
Students’ Perceptions of Learning (SPL)	12	.844
Students’ Perceptions of Teachers (SPT)	11	.842
Students’ Academic Self-Perceptions (SASP)	8	.795
Students’ Perceptions of Atmosphere (SPA)	12	.871
Students’ Social Self-Perceptions (SSSP)	7	.643
DREEM Total Scale	50	.942

Note. Reliability interpretation follows George and Mallery (38): α ≥ .90 = excellent;.80–.89 = good;.70–.79 = acceptable;.60–.69 = questionable; <.60 = poor. DREEM, Dundee Ready Education Environment Measure.

### Measurement invariance

The measurement invariance of the DREEM scale was examined sequentially between two clusters of variables, namely, academic year and gender, according to the model-comparison procedure suggested by Vandenberg and Lance (31) and Putnick and Bornstein (32).

According to the results in Table 3, the configural model showed that the hypothesized factor structure was similar across both academic years and gender groups. In the baseline models, the CFI values were 0.994 and 0.995, the TLI values remained high at 0.993, and the SRMR values were 0.061, indicating a strong incremental fit and acceptable residual fit. However, the RMSEA values were elevated at 0.120 and 0.123 for the year and gender group models, respectively. Thus, the configural models were interpreted as showing acceptable overall fit with caution, rather than full adherence to all conventional absolute fit thresholds (30). Adding equality constraints on factor loadings in the metric model produced only negligible changes in fit for either year (*Δ*CFI = −.001, *Δ*RMSEA =  + .008) or gender (*Δ*CFI = −.001, *Δ*RMSEA =  + .010), and these changes remained within the recommended thresholds of |*Δ*CFI| ≤ .010 (31) and |*Δ*RMSEA| ≤ .015 (32). Additional constraints on item intercepts in the scalar model likewise produced only minimal changes for year (*Δ*CFI =  +.001, *Δ*RMSEA = −.010) and gender (*Δ*CFI =  +.001, *Δ*RMSEA = −.012), again meeting both criteria. Taken together, the stable TLI and SRMR values across model transitions, alongside the negligible *Δ*CFI and *Δ*RMSEA values, support the full scalar invariance of the DREEM scale across both academic year and gender, although the elevated absolute RMSEA values warrant cautious interpretation in subsequent multigroup structural equation modeling.

**Table 3:** Measurement invariance tests across year and gender for the DREEM scale.

Group	Model	*χ* ^2^	df	CFI	RMSEA	SRMR	TLI	*Δ*CFI	*Δ*RMSEA
Year	Configural	9,245.08	2,330	.994	.120	.061	.993	—	—
	Metric	10,330.62	2,375	.993	.128	.064	.992	−.001	+.008
	Scalar	9,690.40	2,517	.994	.118	.060	.993	+.001	−.010
Gender	Configural	9,493.14	2,330	.995	.123	.059	.994	—	—
	Metric	10,971.98	2,375	.994	.133	.063	.993	−.001	+.010
	Scalar	10,047.46	2,517	.995	.121	.058	.994	+.001	−.012

Note. CFI, comparative fit index; RMSEA, root mean square error of approximation; SRMR, standardized root mean square residual; TLI, Tucker–Lewis index; *Δ*CFI, change in CFI from the preceding model; *Δ*RMSEA, change in RMSEA from the preceding model.

### Confirmatory factor analysis

A five-factor CFA model was estimated using WLSMV estimation for all 50 DREEM items. As reported in Table 4, CFI = .996 and TLI = .996, both of which exceeded the.95 threshold, indicating an excellent incremental fit (30). However, the RMSEA was.103 [90% CI (.101,.106)] and SRMR = .086, both slightly above the conventional cutoff values. Taken together, these findings provide mixed but overall supportive evidence for the five-factor structure of the DREEM in this sample population. Given the near-perfect incremental fit indices and the complexity of the 50-item model, the five-factor structure was retained as the theoretically specified representation of DREEM constructs. In interpreting model adequacy, greater weight was placed on the incremental fit indices, while acknowledging that RMSEA values may be inflated in models with large numbers of degrees of freedom (df) (39). Nevertheless, the elevated RMSEA and slightly high SRMR should be acknowledged as limitations when interpreting CFA results.

**Table 4:** Confirmatory factor analysis model fit indices.

Fit Index	Value	Acceptable Threshold
Chi-square (*χ*^2^)	6,279.98	—
Degrees of freedom (df)	1,165	—
*p*-value	<.001	—
CFI	.996	≥.95
TLI	.996	≥.95
RMSEA	.103	≤.08
RMSEA 90% CI Lower	.101	—
RMSEA 90% CI Upper	.106	—
SRMR	.086	≤.08

Note. CFI, Comparative Fit Index; TLI, Tucker–Lewis Index; RMSEA, Root Mean Square Error of Approximation; SRMR, Standardized Root Mean Square Residual. Conventional thresholds were CFI/TLI ≥ .95, RMSEA ≤ .08, and SRMR ≤ .08 (30).

Figure 2 presents the structural equation model examining the associations between the DREEM environmental domains and Students’ Academic Self-Perceptions (SASP). All four domains were significant positive predictors of the SASP. Students’ Perceptions of Learning (SPL) showed the strongest association (*β* = .400, *p* < .001), followed by Students’ Social Self-Perceptions (SSSP) (*β* = .253, *p* < .001), Students’ Perceptions of Atmosphere (SPA) (*β* = .171, *p* = .001), and Students’ Perceptions of Teachers (SPT) (*β* = .148, *p* = .012). These findings indicate that more favorable perceptions of learning, social support, atmosphere, and teaching are associated with higher academic self-perceptions, with perceived learning quality showing the strongest relationship. Together, the four environmental domains accounted for 69% of the variance in SASP (*R*^2^ = .69), indicating that these features of the educational environment are substantially associated with students’ academic self-perceptions.

**Figure 2:**
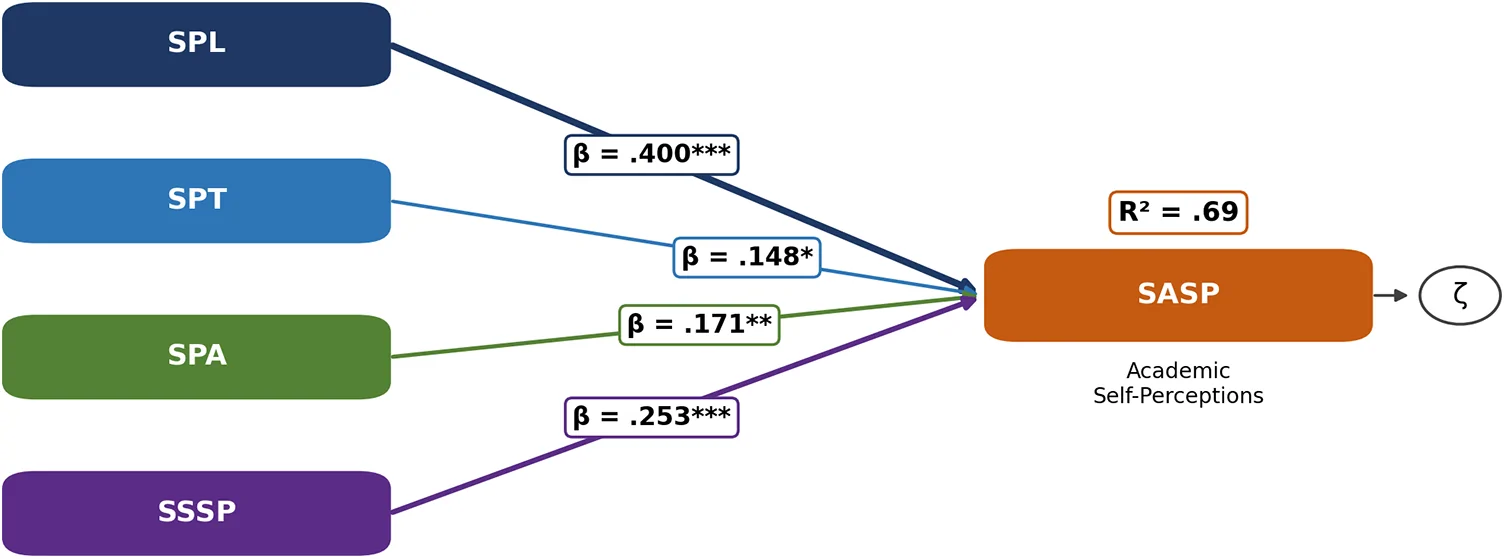
Structural equation model of the associations between educational environment domains and academic self-perceptions. Note. Standardized path coefficients (*β*) are shown on each arrow, and the arrow thickness is proportional to the magnitude of the association. *ζ* denotes the residual variance of SASP, and R^2^ is the proportion of its variance explained. *p < .05, **p < .01, ***p < .001.

### Multigroup SEM: predictors of academic self-perceptions by academic year

Multigroup SEM was used to examine whether SPL, SPT, SPA, and SSSP predicted SASP differently in Years 1 and 2 (Table 5; Figure 3). Among the Year 1 students, SPL (*β* = .583, *p* = .002) and SSSP (*β* = .424, *p* = .002) were significant predictors of SASP, while SPT (*β* = −.073, *p* = .771) and SPA (*β* = .062, *p* = .501) were not. Among Year 2 students, all four domains were statistically significant: SPL (*β* = .421, *p* < .001), SPT (*β* = .285, *p* = .006), SPA (*β* = .176, *p* = .011), and SSSP (*β* = .203, *p* = .006). These findings suggest that as students advance through medical training, a broader set of environmental perceptions becomes predictive of their academic self-concept.

**Table 5:** Multigroup SEM regression coefficients predicting SASP by academic year.

Predictor	B	SE	*z*	*p*	*β*
Year 1					
SPL → SASP	0.435	0.139	3.13	.002**	.583
SPT → SASP	−0.062	0.213	−0.29	.771	−.073
SPA → SASP	0.057	0.085	0.67	.501	.062
SSSP → SASP	0.442	0.140	3.15	.002**	.424
Year 2					
SPL → SASP	0.393	0.091	4.30	<.001**	.421
SPT → SASP	0.251	0.091	2.76	.006**	.285
SPA → SASP	0.184	0.072	2.54	.011*	.176
SSSP → SASP	0.153	0.055	2.76	.006**	.203

Note. B, unstandardized coefficient; SE, standard error; β, fully standardized coefficient. ns, non-significant. *p < .05. **p < .01. ***p < .001.

**Figure 3:**
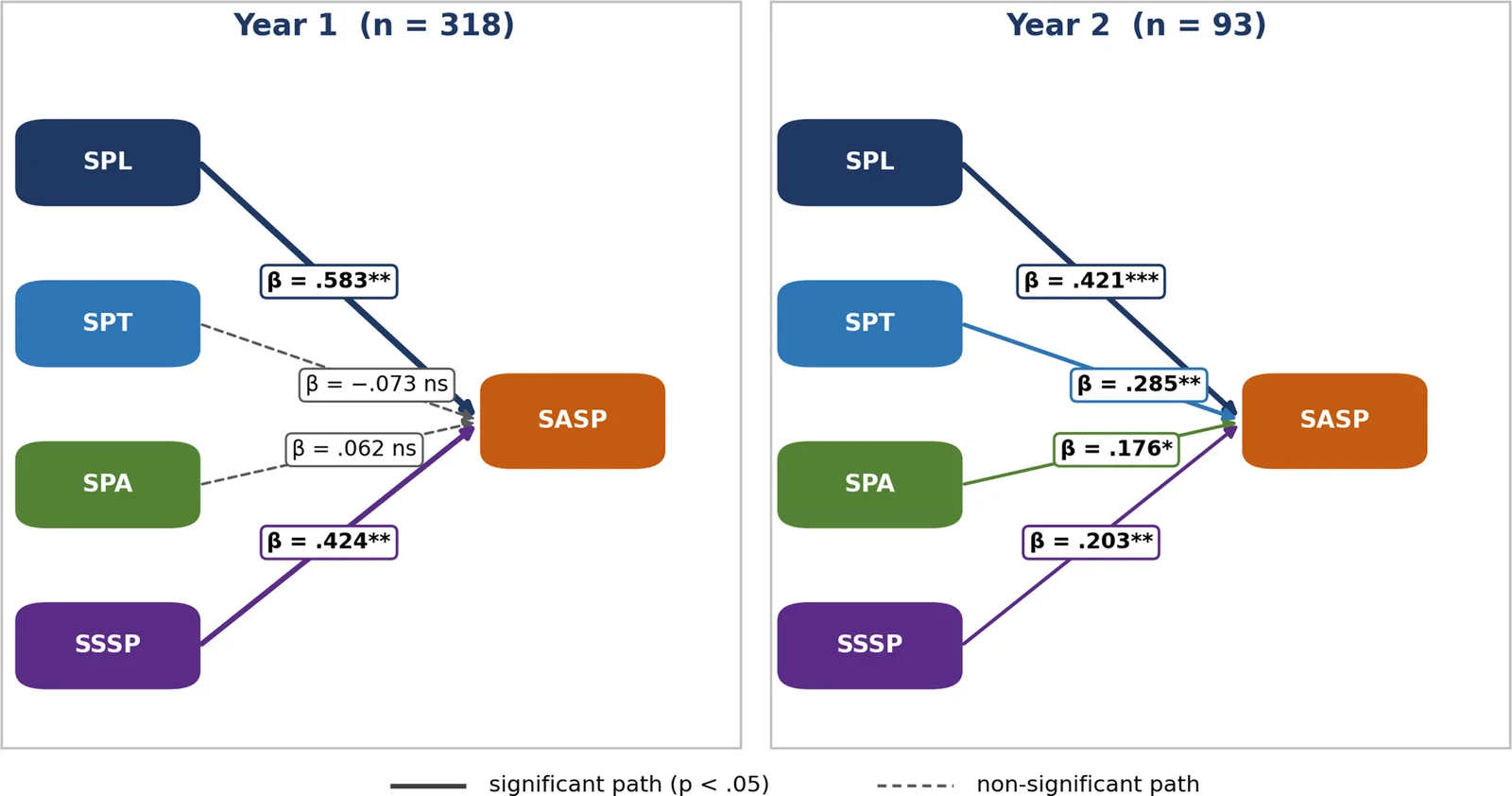
Multigroup structural equation models of academic self-perceptions by academic year. Note. Solid arrows denote statistically significant paths (*p* < .05) and dashed arrows non-significant paths, as indicated in the key beneath the panels; arrow thickness is proportional to the magnitude of association. *β* = standardized coefficient; ns = non-significant. **p < .01, ***p < .001.

### Multigroup SEM: predictors of academic self-perceptions by gender

Gender-based multigroup SEM analyses revealed distinct predictive pathways for male and female students (Table 6; Figure 4). Among male students, SPT (*β* = .409, *p* = .007) and SSSP (*β* = .326, *p* = .001) were significant predictors, whereas SPL (*β* = .232, *p* = .165) and SPA (*β* = .070, *p* = .387) were not. Among female students, SPL was the strongest predictor (*β* = .582, *p* < .001), followed by SPA (*β* = .187, *p* = .020), while SPT (*β* = .015, *p* = .909) and SSSP (*β* = .297, *p* = .094) were not significant. These results indicate gender-differentiated mechanisms through which the educational environment shapes the academic self-concept.

**Table 6:** Multigroup SEM regression coefficients predicting SASP by gender.

Predictor	B	SE	*z*	*p*	β	Sig.
Male students						
SPL → SASP	0.200	0.144	1.39	.165	.232	ns
SPT → SASP	0.361	0.135	2.68	.007	.409	**
SPA → SASP	0.067	0.077	0.87	.387	.070	ns
SSSP → SASP	0.251	0.079	3.20	.001	.326	**
Female students						
SPL → SASP	0.512	0.110	4.64	<.001	.582	***
SPT → SASP	0.013	0.113	0.11	.909	.015	ns
SPA → SASP	0.199	0.086	2.32	.020	.187	*
SSSP → SASP	0.269	0.160	1.68	.094	.297	ns

Note. B, unstandardized coefficient; SE, standard error; β , fully standardized coefficient. ns, non-significant. *p < .05. **p < .01. ***p < .001.

**Figure 4:**
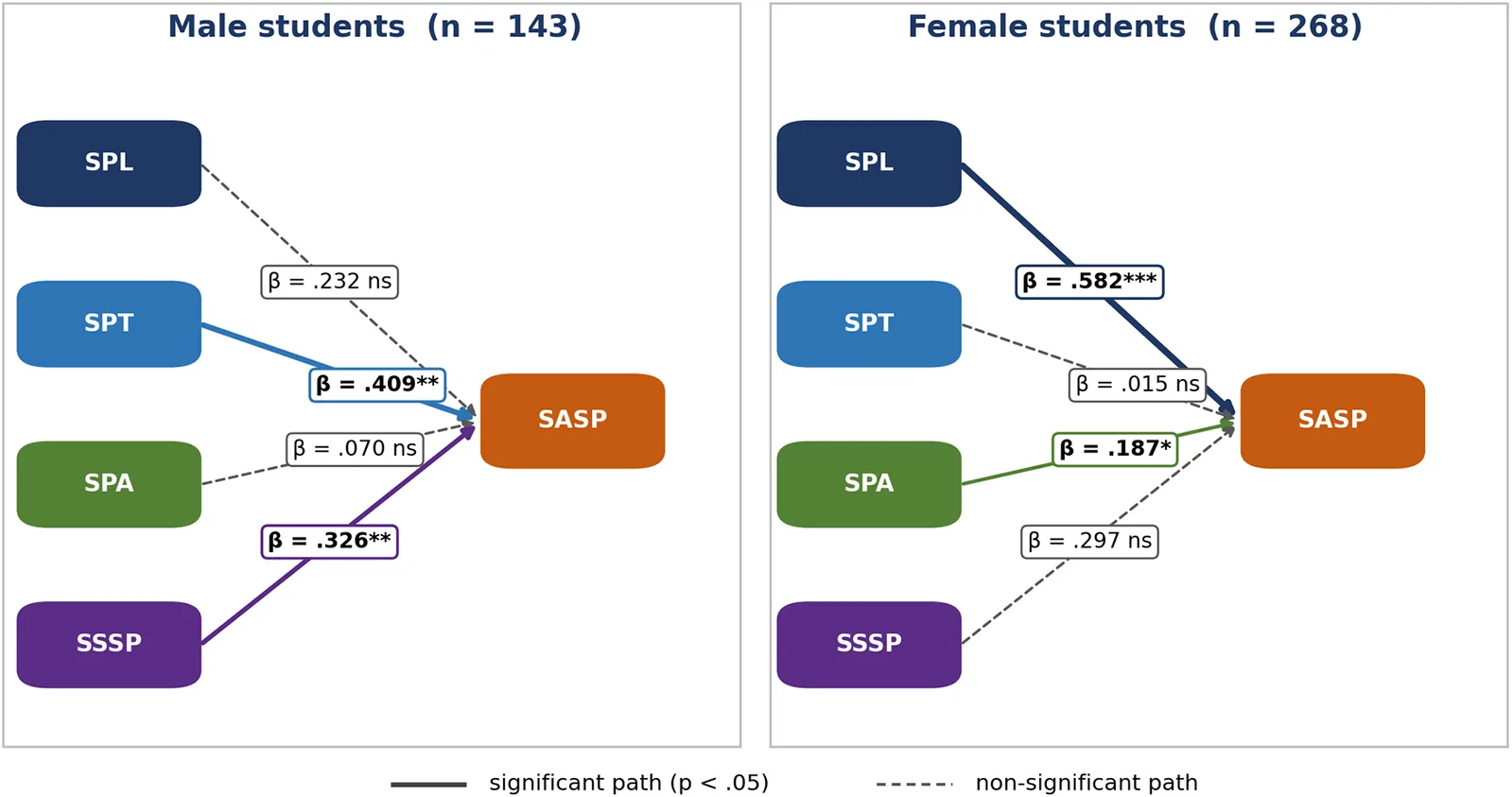
Multigroup structural equation models of academic self-perceptions by gender. Note. Solid arrows denote statistically significant paths (*p* < .05) and dashed arrows non-significant paths, as indicated in the key beneath the panels; arrow thickness is proportional to the magnitude of the association. *β* = standardized coefficient; ns = non-significant. *p < .05, **p < .01, ***p < .001.

### Mediation analysis

Bootstrapped mediation analysis was performed to test whether the SASP mediated the SPL-SPA relationship (Table 7; Figure 5). The total effect of SPL on SPA was significant [*b* = 0.864, 95% CI (0.780, 0.942), *p* < .001]. The indirect effect through SASP [ACME = 0.248, 95% CI (0.162, 0.338), *p* < .001] and the direct effect [ADE = 0.616, 95% CI (0.491, 0.734), *p* < .001] were both significant. The percentage of the total effect mediated by the SASP was 28.7%, indicating a significant indirect association with the SASP. These findings imply that a significant part of the SPL-SPA relationship is mediated by students’ academic self-concept.

**Table 7:** Mediation analysis results: SASP as mediator of the SPL to SPA relationship.

Effect	Estimate	95% CI lower	95% CI upper	*p*
Average Causal Mediation Effect (ACME—Indirect)	0.248	0.162	0.338	<.001***
Average Direct Effect (ADE)	0.616	0.491	0.734	<.001***
Total effect	0.864	0.780	0.942	<.001***
Proportion mediated	0.287	0.187	0.399	<.001***

Note. Mediation was conducted using nonparametric bootstrapping with 5,000 simulations (*N* = 411). ACME, Average Causal Mediation Effect; ADE, Average Direct Effect.

*** *p* < .001.

**Figure 5:**
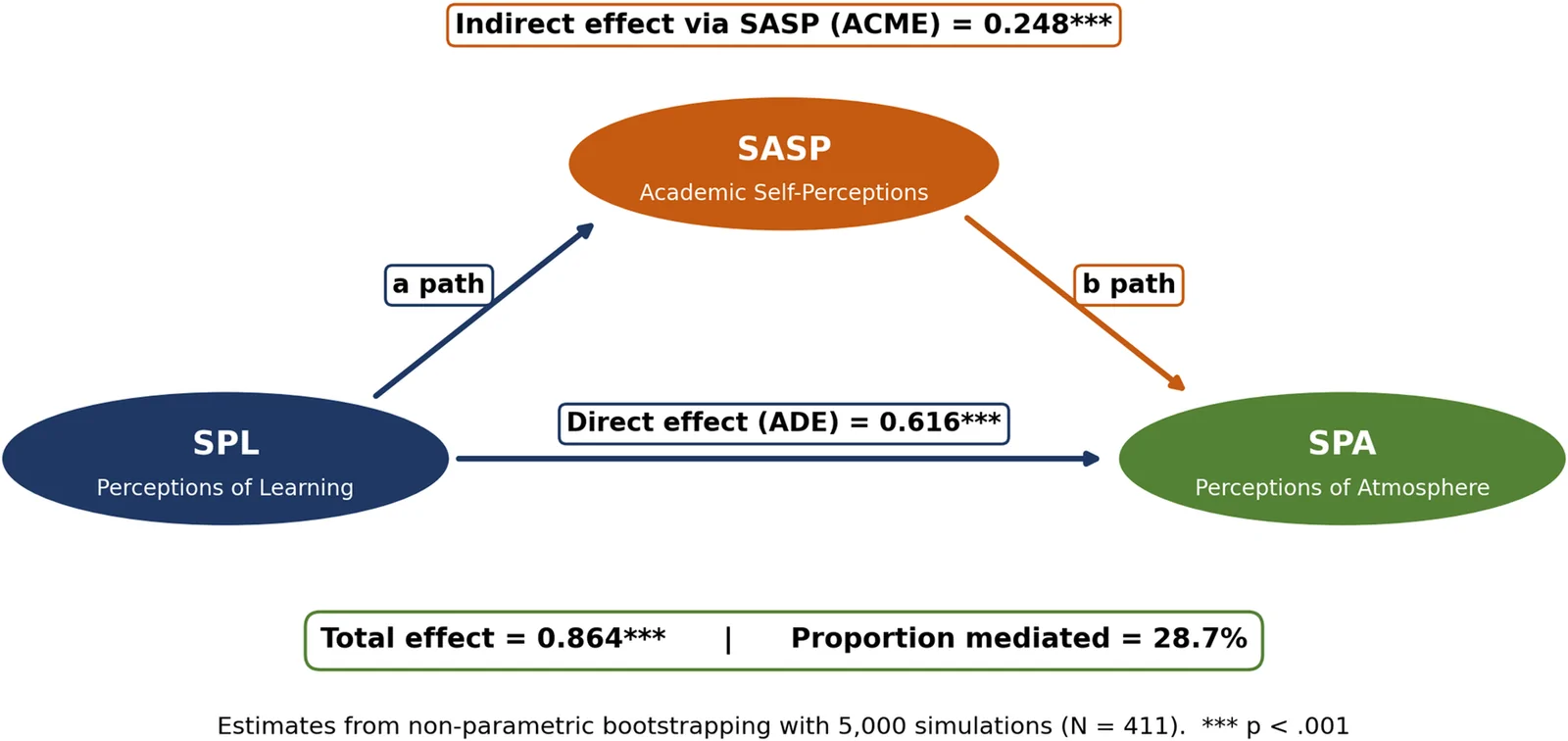
Mediation model of the indirect association between perceptions of learning and perceptions of atmosphere through academic self-perceptions. Note. Paths a and b are the constituent paths of the indirect route through the SASP. ACME, average causal mediation effect (the indirect effect transmitted through SASP); ADE, average direct effect. Estimates were obtained using non-parametric bootstrapping with 5,000 simulations (*N* = 411). ***p < .001.

### Group differences by academic year and gender

Table 8 presents the Wilcoxon rank-sum tests comparing the DREEM subscale and total scores by academic year and gender. Two comparisons reached statistical significance: SPT was higher in Year 1 than in Year 2 (*p* = .033), and SPL was higher among female than male students (*p* = .048). All remaining subscale and total score comparisons were non-significant.

**Table 8:** Wilcoxon rank-sum test results for DREEM subscales by academic year and gender.

Domain	Year W-statistics	Year *p*-value	Gender W-statistics	Gender *p*-value
SPL	15,590	*p* = .426	21,298	*p* = .048*
SPT	16,928	*p* = .033*	19,943	*p* = .427
SASP	13,527	*p* = .210	20,951	*p* = .093
SPA	15,904	*p* = .268	19,659	*p* = .585
SSSP	14,335	*p* = .653	18,556	*p* = .675
DREEM Total	15,744	*p* = .342	20,212	*p* = .304

Note. Wilcoxon rank-sum tests with continuity corrections. W , Wilcoxon statistic.

* *p* < .05.

### Effect sizes

The effect sizes are reported in Table 9. Year-based effects were negligible to small (d range: −0.09 to 0.26), with SPT showing the largest effect size (*d* = 0.26). Gender-based effects were likewise negligible to small (d range: 0.04 to 0.21), the largest being SPT (*d* = 0.21) and SPL (*d* = 0.19), respectively. Rank-biserial correlations were correspondingly small for both academic year (*r* = −0.09 to 0.14) and gender (*r* = −0.03 to 0.11) variables. These values are consistent with the non-significant MANOVA and largely non-significant Wilcoxon results.

**Table 9:** Effect sizes for DREEM subscales by academic year and gender.

Domain	Year d [95% CI]	Year r	Gender d [95% CI]	Gender r
SPL	0.09 [−0.14, 0.32]	0.05	0.19 [−0.01, 0.39]	0.11
SPT	0.26 [0.03, 0.49]	0.14	0.21 [0.00, 0.41]	0.04
SASP	−0.09 [−0.32, 0.15]	−0.09	0.18 [−0.02, 0.39]	0.09
SPA	0.13 [−0.10, 0.37]	0.08	0.10 [−0.10, 0.31]	0.03
SSSP	0.004 [−0.23, 0.24]	−0.03	0.04 [−0.16, 0.25]	−0.03
DREEM Total	0.12 [−0.11, 0.35]	0.06	0.18 [−0.02, 0.38]	0.05

Note. d, Cohen's d computed from the pooled standard deviation, with 95% confidence interval; r, rank-biserial correlation derived from the Wilcoxon rank-sum statistic. Positive values indicate higher scores in Year 1 (academic year comparison) and female students (gender comparison). Effect size interpretation: negligible < 0.20; small = 0.20–0.49; medium = 0.50–0.79; large ≥ 0.80 (40).

**Table 10:** MANOVA results for DREEM subscales by academic year and gender.

Effect	Pillai's Trace	F	df	*p* (Interpretation)
Academic Year	0.025	2.07	5	*p* = .069 (ns)
Gender	0.016	1.28	5	*p* = .269 (ns)
Residuals	—	—	408	—

Note. MANOVA was conducted using Pillai's trace criterion with all five DREEM subscale totals as the multivariate outcome. ns, non-significant (*p* > .05).

### Multivariate analysis of variance

A MANOVA using Pillai's trace examined the simultaneous effects of academic year and gender on the five DREEM subscales (Table 10). Neither academic year, Pillai's trace = .025, F(5, 404) = 2.07, *p* = .069, nor gender, Pillai's trace = .016, F(5, 404) = 1.28, *p* = .269, produced significant multivariate effects. These results indicate that the observed univariate differences are domain-specific rather than indicative of broad systematic differences in educational climate perceptions.

## Discussion

The findings suggest that students may differ in how dimensions of the educational environment relate to academic self-perceptions, even when broad mean-level differences between groups are limited. This distinction is particularly relevant in TBL-oriented medical education, where perceptions of learning, teaching, peer interaction and atmosphere are closely interconnected. The findings are discussed below in relation to the psychometric properties of the DREEM and the structural relationships observed across academic years and gender.

The CFA provided mixed but generally supportive evidence for the original five-factor structure of the DREEM. The CFI and TLI were both.996, exceeding the.95 criterion (30), whereas the RMSEA (.103) was above the conventional.08 threshold. This elevation may partly reflect the behavior of the RMSEA in large ordinal models estimated with WLSMV (39). Although a validation of the Polish version of the DREEM in dental students reported a lower RMSEA (.043) (41), direct comparison is limited by differences in the estimation approach and study population. Therefore, the five-factor structure was retained, while the elevated RMSEA warrants cautious interpretation.

Within Kane's (25) argument-based validity framework, the CFA provides evidence regarding internal structure, whereas multigroup SEM and mediation analyses contribute evidence concerning relationships among DREEM domains and SASP. These findings support a partial validity argument for the subscale-level interpretation of the DREEM in this educational context, although generalizability and consequential evidence were not examined.

The total DREEM demonstrated excellent internal consistency (*α* = .942), whereas the SSSP showed the lowest reliability (*α* = .643), consistent with previous studies (14, 37). Its lower reliability may partly reflect the heterogeneity of items addressing social life, loneliness, and accommodation (37). This finding suggests that the SSSP should be interpreted with greater caution than the other subscales.

Multigroup SEM identified different predictors of SASP across the academic years. In Year 1, SPL and SSSP were significant predictors, a pattern consistent with Bandura's (42) self-efficacy framework. Within the TBL curriculum, repeated readiness assurance, peer discussion, and collaborative learning may provide mastery and social experiences that contribute to academic self-perception (3). The non-significant SPT pathway may also reflect the facilitator-centered rather than teacher-centered role characteristic of TBL. Among Year 2 students, all four environmental domains were associated with SASP, compared with SPL and SSSP in Year 1. This broader pattern may reflect differences in curriculum format, academic stage, or block-specific cognitive demands (43), which cannot be separated in the present cross-sectional study. This finding is consistent with previous DREEM research, suggesting greater differentiation in educational environment perceptions across stages of training (19, 21). However, given the smaller Year 2 sample, these subgroup-specific associations should be interpreted as context-dependent findings that require replication in larger cohorts.

Gender-stratified analyses revealed distinct structural pathways. Among male students, teacher quality (SPT) and peer social dynamics (SSSP) were significant predictors of SASP; among female students, perceived learning quality (SPL) and institutional atmosphere (SPA) were significant. This differential pattern can be interpreted through Bandura's (42) sources of self-efficacy: female students appear to weight enactive mastery experiences as the primary self-efficacy signal, whereas male students rely more heavily on social persuasion and vicarious experience channels. The structural differentiation observed here cannot be detected by mean-comparison methods, which accounts for the inconsistent gender findings across prior DREEM studies that relied solely on t-tests and ANOVA (12, 17). The Wilcoxon results confirmed that female students scored significantly higher on SPL (*p* = .048), and the structural analysis extended this by demonstrating that SPL is the structural foundation of their academic self-concept in this curriculum.

The SPL–SPA relationship was partially mediated by SASP, with 28.7% of the total effect conveyed through academic self-perceptions. This pattern is consistent with Bandura's (42) self-efficacy theory, which states that effective learning experiences may strengthen academic self-beliefs that, in turn, shape perceptions of the broader educational atmosphere. TBL readiness assurance may support this process by providing repeated opportunities for students to calibrate their academic confidence against their peers and facilitators’ feedback (4). The remaining direct effect indicates that SPL may also be related to SPA through pathways independent of the SASP. Academic self-perception has also been identified as one of the two strongest DREEM domain correlates of student thriving, alongside social self-perception, in a multinational sample (44). Given the cross-sectional design, this finding should be interpreted as an indirect structural association, rather than a causal sequence.

Neither academic year nor gender produced significant multivariate differences in DREEM subscale scores (year, *p* = .069; gender, *p* = .269), and year-based effect sizes were negligible to small. However, the SEM analyses identified different structural relationships across the year and gender groups. This distinction suggests that similar mean DREEM scores may coexist with different patterns of association among the educational environment domains. Accordingly, reliance on mean comparisons alone may overlook subgroup-specific relationships relevant to academic self-perception.

### Implications

The findings have four practical implications for TBL-based medical curricula. **First**, the relatively low SSSP scores suggest that participation in formal teams does not necessarily ensure social connectedness. Consistent with Wenger's (45) community of practice framework, structured team-building and psychological safety initiatives may complement TBL by strengthening students’ sense of belonging.

Second, gender-differentiated structural pathways suggest that environmental support may need to consider subgroup-specific patterns. Faculty-student relationships and peer dynamics may be particularly relevant to male students’ academic confidence, whereas learning quality and institutional atmosphere may be more salient for female students. These patterns are specific to the current institutional context and require replication elsewhere.

Third, the partial mediation of the SPL–SPA relationship through SASP suggests that perceived learning quality may influence students’ perceptions of the broader educational atmosphere, partly through academic self-perceptions. Strengthening the clarity, quality, and formative feedback of TBL activities may therefore support both academic confidence and perceptions of the learning environment.

Fourth, the differences between Year 1 and Year 2 indicate that educational environment monitoring may benefit from a year-specific approach. Year 1 findings emphasized learning quality and peer integration, whereas Year 2 findings also involved teaching quality and atmosphere. Periodic subscale-level DREEM assessments may help institutions identify cohort-specific areas for curricular and student-support improvement.

### Limitations

This study had several limitations. First, the cross-sectional design precluded causal inference. The observed associations between environmental perceptions and academic self-concept do not establish directionality, and the mediation findings represent plausible structural pathways rather than definitive causal mechanisms.

Second, the sample was restricted to Year 1 and Year 2 preclinical students at a single institution. Although the overall response rate was 58.7%, voluntary participation introduces a potential non-response bias. If participants differed systematically from non-participants in their perceptions of the educational environment, the observed DREEM estimates and structural associations may not fully represent the eligible population. As respondents and non-respondents were not directly compared, the magnitude of this bias is unknown.

Third, the absolute sample sizes and response rates varied between cohorts. Year 1 yielded a 64.1% response rate (*n* = 318) compared to 45.6% for Year 2 (*n* = 93), resulting in a wider margin of error for the Year 2 subgroup analyses (±7.5% vs. ± 3.3%). This smaller Year 2 subgroup may reduce the precision and stability of the parameter estimates; thus, year-stratified SEM findings require cautious interpretation and future replication. No *a priori* power analysis was performed because the entire eligible population was invited rather than a calculated sample. Based on the standard errors obtained, the Year 2 model had approximately 80% power to detect standardized path coefficients of approximately.27 or larger: the SPL pathway (*β* = .421) therefore rests on adequate precision, whereas the smaller SPT, SSSP, and SPA pathways (*β* = .285,.203, and.176) fall at or below this threshold and should be regarded as provisional pending replication. The margin-of-error estimates reported above describe the precision of the population proportion estimates and do not index the statistical power of the structural models.

Fourth, comparing Year 1 and Year 2 confounds the academic year, pedagogical format (TBL vs. TBL + PBL), and block content (GIT vs. neuroscience), preventing the isolation of these variables. Additionally, the lack of a lecture-based comparator means that the observed structural pathways cannot be exclusively attributed to the TBL design. Nevertheless, the structural SEM approach demonstrated here is transferable to other institutions using the DREEM inventory to examine subgroup pathway differences.

Fifth, because the DREEM subscales are summed ordinal scores comprising different numbers of items, effect sizes are reported both as Cohen's d and as rank-biserial correlations. The two indices were concordant in this sample, but rank-biserial r is the more appropriate measure for non-parametric comparisons and is recommended for future studies using ordinal DREEM data. Furthermore, while measurement invariance across academic year and gender was formally assessed and change indices supported configural, metric, and scalar invariance, the absolute RMSEA values for the invariance models remained high. Therefore, cross-group comparisons of structural paths should be replicated in independent samples.

Finally, all DREEM data relied on self-reported perceptual measurements. While self-report is appropriate for assessing subjective student perceptions, relying on a single instrument introduces the risk of common method variance.

### Future research directions

Several directions for future research emerge from these findings. A longitudinal design following the same students from Year 1 through clinical placements would yield better evidence regarding how structural predictor patterns shift across curriculum formats and across the transition from TBL-dominant preclinical training to supervisor-dependent clinical rotations, where the efficacy source structure is likely to change. Since Years 1, 2, and 3 at Alfaisal represent a natural pedagogical gradient–pure TBL, TBL + PBL hybrid, and PBL-only, respectively–a three-cohort structural comparison within the same institution would offer a well-controlled test of whether the Year 1 dominance of SPL and SSSP and the non-significance of SPT are specific to TBL's de-centered facilitation model. Under PBL, where the tutorial facilitator is the primary driver of case discussion quality, Bandura's (42) social persuasion source of efficacy predicts that SPT should re-emerge as a dominant predictor of SASP—a hypothesis that this study cannot test with only two cohorts, but that the institution is structurally positioned to examine in future work. Future work should also examine whether the gender-differentiated predictive structures replicate across different institutional and national contexts and whether they hold in gender-segregated vs. co-educational settings. Given that the present data were collected during specific academic blocks—GIT for Year 1 and Neuroscience for Year 2—future multi-block surveys of the same cohort would allow separation of block-specific cognitive load effects from year-level and curriculum format effects, addressing the three-variable confound identified in the Limitations section.

## Conclusion

This study confirms the five-factor structure of the DREEM in a TBL-based preclinical medical curriculum and demonstrates that subgroup differences in educational environment experience reside primarily in structural relationships among perceptions rather than in mean score levels. The distinct predictive pathways by academic year and gender, the partial mediation of the SPL–SPA relationship through academic self-perceptions, and the persistently lower social self-perception scores despite an explicitly team-based curriculum together form a more differentiated picture of how TBL students experience their learning environment than mean-level analyses alone can provide.

For institutions using TBL, the findings confirm the value of periodic subscale-level environmental assessment and demonstrate that effective learning experiences, social processes, and teaching quality have population-specific roles in shaping students’ academic confidence. These roles are structured differently by year of training, curriculum format, and gender in ways that aggregate scores cannot reveal. Grounded in Bandura's self-efficacy theory, the results suggest that investment in perceived learning quality generates returns across multiple environmental domains via academic self-concept, a mechanism that TBL's readiness assurance cycle is structurally positioned to activate. Sustained, structurally informed use of validated instruments, such as the DREEM, is essential to realize this potential.

## Data Availability

The raw data supporting the conclusions of this article will be made available by the authors, without undue reservation.
